# List of reviewers 2022

**DOI:** 10.1192/bji.2023.7

**Published:** 2023-05

On behalf of the editorial board, the Editor-in-Chief would like to thank the following for their contribution as peer reviewers in the period from January 2022 – December 2022. Their anonymous work for the journal forms the foundation to its success, and is highly appreciated.

Karim Abdel Aziz

Ahmed Abdelgawad

Nadim Almoshmosh

Rakesh Chadda

Kathy Chatzigeorgiou

Anna Datta

Victoria Patricia Dela Llana

Hine Elder

Md. Omar Faruk

Sandeep Grover

Essam Hassan

Margaret Hogan

Sahadat Hossain

Guesche Huebner

Mark Kaggwa

Yasser Khazaal

Konstantinos Kotsis

Nick Kowalenko

Mark Lawrence

Konstantina Magklara

Alessandro Massazza

Rosalind McAlpine

Giulia Piazza

Felipe Picon

Mariana Pinto da Costa

Hashim Reza

Paul Robertson

Stella Rosson

Sheikh Shoib

Pau Soldevila-Matías

Stephen Stathis

Chathurie Suraweera

Sirirat Ularntinon

James Woollard

Ziyan Xu

